# Progressive multifocal cerebral infarction in a young kidney transplant recipient due to thrombotic microangiopathy

**DOI:** 10.1186/1471-2369-15-59

**Published:** 2014-04-07

**Authors:** Arash Haghikia, Meike Heeren, Clemens Bockmeyer, Bernd Haubitz, Wilfried Gwinner

**Affiliations:** 1 Center of Internal Medicine, Hannover Medical School, Hannover, Germany; 2 Department of Cardiology and Angiology, Hannover Medical School, Hannover, Germany; 3 Department of Neurology, Hannover Medical School, Hannover, Germany; 4 Department of Pathology, Hannover Medical School, Hannover, Germany; 5 Department of Neuroradiology, Hannover Medical School, Hannover, Germany; 6 Department of Nephrology and Hypertensiology, Hannover Medical School, Hannover, Germany

**Keywords:** Cerebral infarctions, Kidney transplantation, Microangiopathy

## Abstract

**Background:**

Renal transplant recipients frequently experience neurological complications. Whereas ischemic stroke, cerebral haemorrhage or hypertensive encephalopathy often result from vascular alterations prior to transplantation, other cerebral diseases like CNS infections, primary brain tumors and drug induced neurotoxicity may develop as consequences of the required post-transplant immunosuppressive treatment.

**Case presentation:**

Here we report on an unusual clinical course of a young kidney transplant recipient with a cluster of fulminant necrotic brain lesions within a period of two months due to thrombotic microangiopathy.

**Conclusion:**

Cerebral ischemia in organ transplant recipients should prompt one to consider thrombotic microangiopathy.

## Background

Neurological complications post transplantation include infections and tumours promoted by the immunosuppressive therapy in general and more frequently, tremor and peripheral neuropathies which are commonly related to the therapy with calcineurin inhibitors [1]. Severe calcineurin inhibitor-related side effects occur in 10% and are, mostly, reversible after dose reduction or cessation of the drug. These include decreased responsiveness, hallucinations, delusions, seizures, cortical blindness, and stroke-like episodes [2]. Rarely, calcineurin inhibitor related neurotoxicity presents as so-called “reversible posterior leukoencephalopathy” (RLPS) [3].

## Case presentation

A 25-year-old male caucasian patient presented with a 1-week history of left-sided weakness, preceded by general fatigue and progressive forgetfulness in the previous two months. His medical history comprised a kidney transplantation 12 years earlier for end-stage renal failure due to focal and segmental glomerulosclerosis, a longstanding well-controlled hypertension (RR 130/85 mmHg in the previous months), mild pancytopenia with a previous diagnosis of a hypoplastic bone marrow with presumed toxic cause. At admission, his therapy included cyclosporine A 35 mg b.i.d, prednisolone 7.5 mg, valsartan 160 mg b.i.d and 40 μg darbepoetin alfa every two weeks. Arterial blood pressure at admission was 129/90 mmHg and body temperature was normal. The strength of the left-sided limbs was mildly decreased (4+/5). Initially, leucocytopenia and mild thrombopenia, a haemoglobin concentration of 11 g/dl and normal C-reactive protein were present (Table 1). The serum creatinine concentration was 156 μmol/l (equaling an eGFR of 47 ml/min) which was in line with values of the preceding years. Actual and previous values for lactate dehydrogenase were normal (238 U/l).

**Table 1:** Depicts the course of some laboratory values

**Lab data**	**4 Weeks earlier**	**Admission**	**Shortly before death**
Hemoglobin (g/dl)	11	11.1	8.8
White blood cells (thousand/μl)	3.9	0.7	4.0
Platelets (thousand/μl)	168	265	15
Creatinine (μmol/l)	156	161	124
CRP (mg/l)	6	4	4
LDH (U/l)	238	198	601
Cyclosporin A level (μg/l).	64	< 15	<15

**Figure 1:**
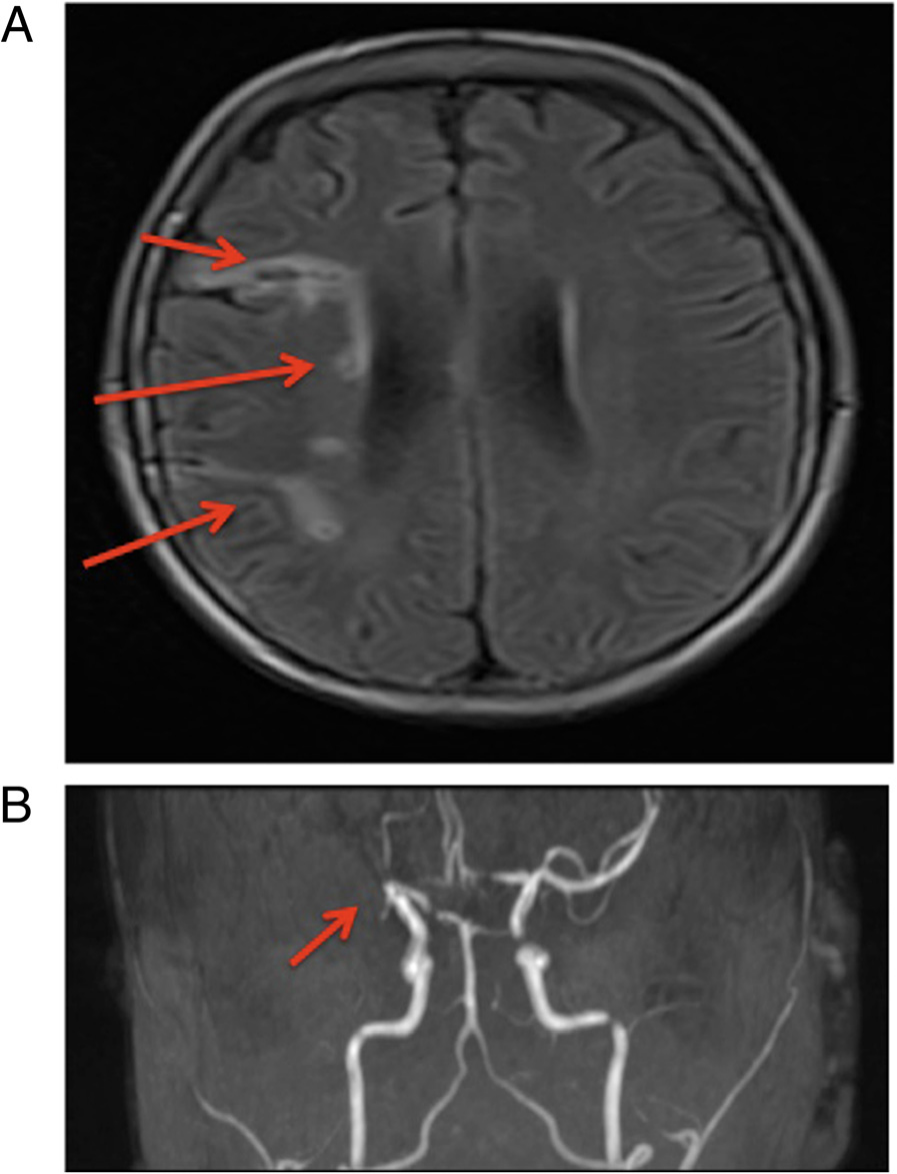
**Cranial MR Imaging of the cerebral lesions. (A)** Initial cranial MRI demonstrating right-sided temporo-parietal and thalamic lesions of different age. **(B)** MR angiography depicting missing flow signal of the right middle cerebral artery.

A cranial MRI showed right-sided temporo-parietal and thalamic lesions (Figure1A). Correspondingly, MR angiography revealed a missing flow signal of the right middle cerebral artery (Figure 1B). Cardiac thromboembolism was excluded by transesophageal echocardiography. By Doppler ultrasonography and MR angiography, arterial occlusive disease, vasculitis and aneurysms of the extracranial brain-supplying arteries and of the aorta were excluded. Vasculitits was further excluded by negative results for anti-nuclear antibodies, ANCA, anti-mitochondrial antibodies, anti-cardiolipin antibodies, cryoglobulins/HCV and HIV status. Acetyl salicylic acid was prescribed and the patient was discharged.

Four weeks later he was admitted again because of listlessness and mutism. At this admission leucopenia had progressed to 0.7 thousand/μl, the haemoglobin concentration was 11.1 g/dl and the thrombocyte count 200 thousand/μl. The serum creatinine concentration was 161 μmol/l and the cyclosporine A trough level (measured by mass spectrometry; LC-MS/MS) was below the detection limit (15 μg/l) (Table 1). A bone marrow examination revealed hypoplasia with dysmature haematopoiesis. An electroencephalogram displayed left-sided fronto-temporal intermittent rhythmic delta-activity without epileptiform discharges. The cerebrospinal fluid (CSF) was normal including virology (CMV, HSV, VZV, EBV, enterovirus and JCV). Moreover, repeatedly negative results of CRP and normal body temperature argued against an infection.

**Figure 2:**
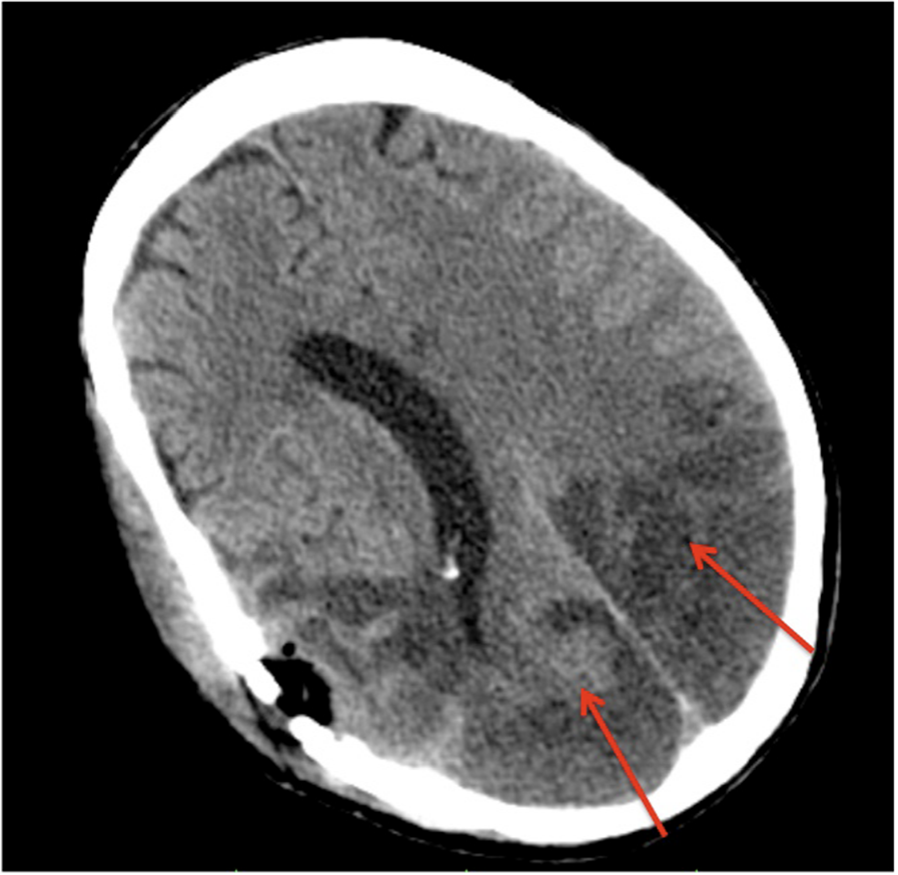
A cranial CT-scan at a later stage of disease demonstrated additional bilateral infarct lesions in the posterior circulation territory.

**Figure 3:**
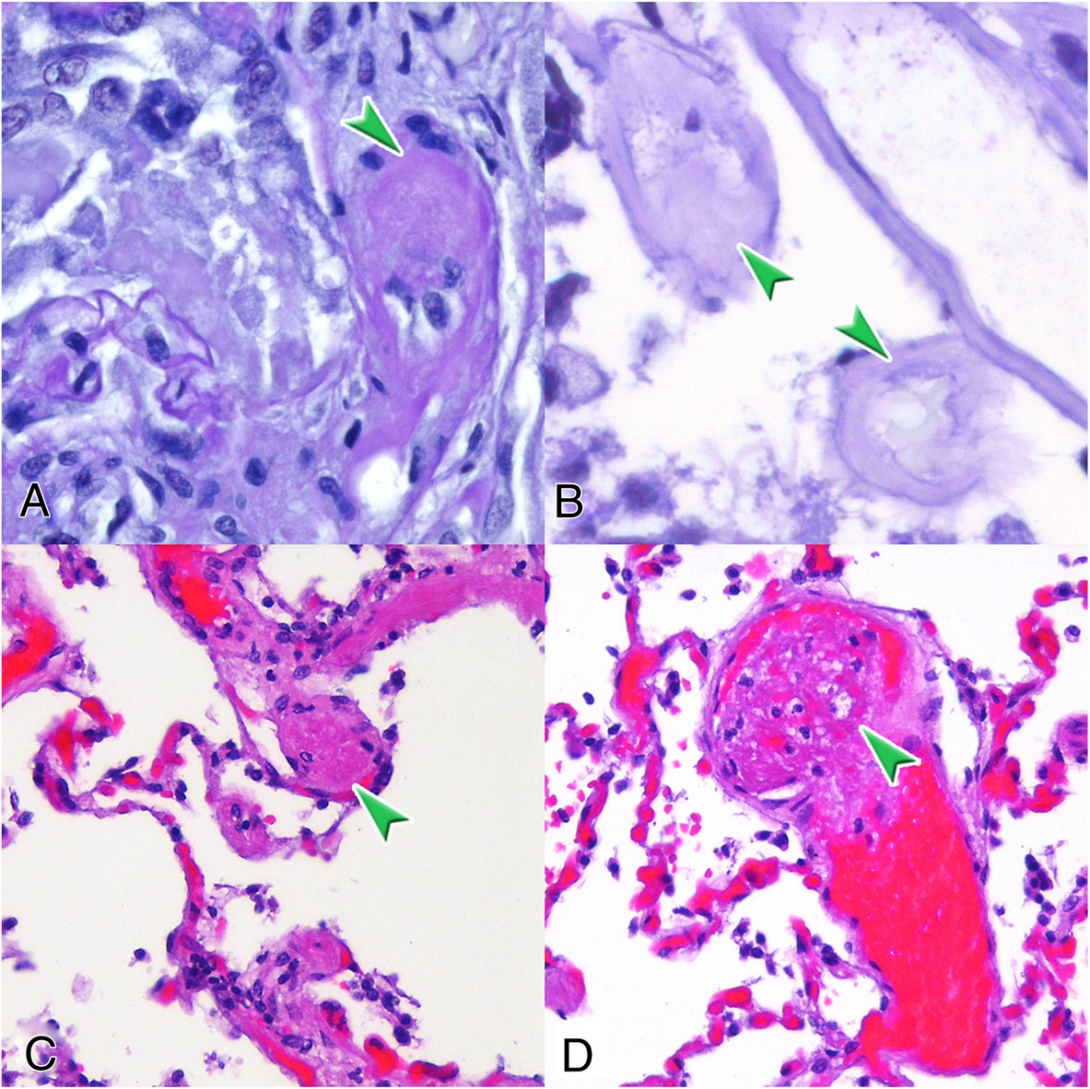
**Postmortem diagnosis of thrombotic microangiopathy.** Intravascular thrombi are indicated by arrows. **A**: pre-glomerular arteriole of the transplanted kidney; **B**: arteriole in the brain tissue; and thombi in a smaller **(C)** and in a larger arterial **(D)** vessel of the lung. PAS staining **(A, B)**, HE staining **(C, D)**, magnification x400.

At the 5^th^ day, he was discovered having bilateral blindness accompanied by moderate to severe loss of conscious (Glascow coma scale of 8) within the next two days. At the onset of these symptoms cyclosporine A was paused. A follow-up cranial MRI revealed new ischemic lesions of the left-sided thalamus and both occipital regions. Brain biopsy was decided and the histology showed extensive necrosis and arteriolar hyalinosis. No findings of vasculitis, inflammatory, infectious (negative tests for CMV, HSV, VZV, EBV and JCV) or neoplastic processes were detected. In the next two days the patient increasingly developed severe hypertension with systolic values above 220 mmHg. Three days after the brain biopsy thrombocyte count rapidly declined to 25 thousand/μl. Schistocytes remained negative and ADAMTS-13 activity and C3c, C4 and CH50 complement titers were normal but lactate dehydrogenase increased to 550 U/l. A cranial CT-scan (Figure 2) demonstrated bilateral infarct lesions in the posterior circulation territory. The patient was transferred for anti-hypertensive treatment to the ICU, where he died after developing further cerebral infarctions of the brain stem. Post-mortem examination revealed disseminated thrombotic microangiopathy in the brain, the lungs and the renal allograft (Figure 3).

## Conclusions and consent

The prevalence of acute cerebral ischemia among renal transplant recipients has been estimated at 8% [4]. Arteriosclerotic vascular disease or hypertensive encephalopathy was unlikely causative in this young patient because of the absence of relevant risk factors. Also, arterial thromboembolism was definitely ruled out. CNS infection which comprise about 40% of all cerebral complications and are mostly seen during the first 24 months post transplantation [5], was not evident in our patient Although calcineurin inhibitors have emerged as a frequent cause of neurologic symptoms and disease, affecting 25% to 59% of transplant patients, gross infarction is typically not seen with these compounds [2]. In our patient, previous cyclosporine trough levels were consistently very low and no clinical improvement was achieved after stopping cyclosporine A treatment. Nevertheless, this does not completely argue against cyclosporine A-induced vascular injury because high calcineurin-inhibitor levels appear to be not a pre-requisite and cessation of the drug in our patient may have been too late to change the course of disease.

Another complication which may be attributed to cyclosporine A is thrombotic microangiopathy (TMA) [6]. A study by Zarifian et al. reviewing 188 patients reported TMA as the cause of renal graft dysfunction in 14% of renal graft recipients and 92% of the TMA cases were on therapy with cyclosporine A [7]. Noteworthy, conversion from cyclosporine A to tacrolimus resulted in salvage of graft function in 81% of the cases. Onset of TMA was highly variable with 4 days to 2190 days post-transplantation, suggesting that other precipitating factors besides cyclosporine A may have been present in some patients. In none of the patients, extrarenal involvement of TMA was reported. In our case, postmortal histopathological examination clearly showed signs of TMA in the brain, in the lungs and in the renal transplant. Early recognition of TMA in our patient was impeded as the initial biopsy did not report TMA as the cause of brain necrosis, mild pancytopenia was pre-existing and clear signs of thrombotic microangiopathy with hemolysis were missing until major cerebral damage had occurred. Also, in our patient there was no clue for TMA in the former history with regard to his original renal disease, in an allograft biopsy performed two years before his death, or in his family. As diarrhoea was not present at any time we did not include infectious haemolytic uremic syndrome in our differential diagnosis. Unfortunately, we could not perform thorough complement gene analysis in this short disease course to establish whether atypical haemolytic uremic syndrome was causative for the TMA. Nevertheless, C3c, C4 and CH50 complement titers were normal at the time of presentation.

This case emphasizes that TMA can take a smouldering course in transplant patients without typical signs like severe thrombopenia or the characteristic picture of haemolytic-uremic syndrome with severe anemia and renal dysfunction. Earlier intervention may have had changed the course in our patient. Although there is no proof that cyclosporine A was causative, prompt withdrawal may have been beneficial. Plasmapheresis has been the mainstay in the treatment of TMA. Improved allograft outcomes have been reported particularly with pre-emptive plasmapheresis therapy in patients with known risk for atypical haemolytic uremic syndrome [8]. More recently, eculizumab has emerged as an efficacious therapy in such patients, either with or without plasmapheresis treatment [9]. Yet, at the point where TMA with a possible underlying diagnosis of atypical haemolytic uremic syndrome had to be considered in our patient, the cerebral infarction was advanced excluding a relevant improvement by this therapy.

Written informed consent was from the parents of the patient for the publication of this report and any accompanying images.

## Competing interests

The authors declare that they have no competing interests.

## Authors’ contributions

AH and WG were involved in the treatment of the patient and acquisition, analysis and interpretation of the medical history of the patient, drafting the manuscript and have given final approval of the version to be published. MH, BH and CB were involved in the diagnostic procedures, interpretation and discussing the results, and in critically revising the manuscript for important intellectual content. All authors read and approved the final manuscript.

## Pre-publication history

The pre-publication history for this paper can be accessed here:

http://www.biomedcentral.com/1471-2369/15/59/prepub
